# Enhancement of HIV-1 infection and intestinal CD4+ T cell depletion ex vivo by gut microbes altered during chronic HIV-1 infection

**DOI:** 10.1186/s12977-016-0237-1

**Published:** 2016-01-14

**Authors:** Stephanie M. Dillon, Eric J. Lee, Andrew M. Donovan, Kejun Guo, Michael S. Harper, Daniel N. Frank, Martin D. McCarter, Mario L. Santiago, Cara C. Wilson

**Affiliations:** Department of Medicine, University of Colorado Anschutz Medical Campus, Aurora, CO USA; University of Colorado Microbiome Research Consortium, Aurora, CO USA; Department of Surgery, University of Colorado Anschutz Medical Campus, Aurora, CO USA

**Keywords:** Human immunodeficiency virus, Microbiome, Microbial translocation, Gut, Lamina propria CD4 T cells, Gram-negative bacteria, Lipopolysaccharide

## Abstract

**Background:**

Early HIV-1 infection is characterized by high levels of HIV-1 replication and substantial CD4 T cell depletion in the intestinal mucosa, intestinal epithelial barrier breakdown, and microbial translocation. HIV-1-induced disruption of intestinal homeostasis has also been associated with changes in the intestinal microbiome that are linked to mucosal and systemic immune activation. In this study, we investigated the impact of representative bacterial species that were altered in the colonic mucosa of viremic HIV-1 infected individuals (HIV-altered mucosal bacteria; HAMB) on intestinal CD4 T cell function, infection by HIV-1, and survival in vitro. Lamina propria (LP) mononuclear cells were infected with CCR5-tropic HIV-1_BaL_ or mock infected, exposed to high (3 gram-negative) or low (2 gram-positive) abundance HAMB or control gram-negative *Escherichia coli* and levels of productive HIV-1 infection and CD4 T cell depletion assessed. HAMB-associated changes in LP CD4 T cell activation, proliferation and HIV-1 co-receptor expression were also evaluated.

**Results:**

The majority of HAMB increased HIV-1 infection and depletion of LP CD4 T cells, but gram-negative HAMB enhanced CD4 T cell infection to a greater degree than gram-positive HAMB. Most gram-negative HAMB enhanced T cell infection to levels similar to that induced by gram-negative *E. coli* despite lower induction of T cell activation and proliferation by HAMB. Both gram-negative HAMB and *E. coli* significantly increased expression of HIV-1 co-receptor CCR5 on LP CD4 T cells. Lipopolysaccharide, a gram-negative bacteria cell wall component, up-regulated CCR5 expression on LP CD4 T cells whereas gram-positive cell wall lipoteichoic acid did not. Upregulation of CCR5 by gram-negative HAMB was largely abrogated in CD4 T cell-enriched cultures suggesting an indirect mode of stimulation.

**Conclusions:**

Gram-negative commensal bacteria that are altered in abundance in the colonic mucosa of HIV-1 infected individuals have the capacity to enhance CCR5-tropic HIV-1 productive infection and depletion of LP CD4 T cells in vitro. Enhanced infection appears to be primarily mediated indirectly through increased expression of CCR5 on LP CD4 T cells without concomitant large scale T cell activation. This represents a novel mechanism potentially linking intestinal dysbiosis to HIV-1 mucosal pathogenesis.

**Electronic supplementary material:**

The online version of this article (doi:10.1186/s12977-016-0237-1) contains supplementary material, which is available to authorized users.

## Background

Human immunodeficiency virus (HIV)-1 disease is associated with extensive structural, immunological and microbial alterations in the intestinal microenvironment which are thought to contribute to HIV-associated chronic immune activation and HIV-1 disease progression. Early studies utilizing the non-human primate simian immunodeficiency virus (SIV) animal model of HIV-1 infection demonstrated that high levels of viral replication and massive CD4 T cell depletion occurred in the intestine as early as 7 days post-infection [1–3]. In HIV and SIV infection, extensive depletion of gut T helper 17 (Th17) and Th22 cells is associated with pathogenesis [4–8]. In contrast to peripheral CD4 T cells, reconstitution of intestinal CD4 T cells following anti-retroviral therapy (ART) is typically delayed and in many individuals, reconstitution is incomplete and dependent upon time of ART initiation (reviewed in [9]). The immense levels of viral replication that occur in the gastro-intestinal (GI) tract are likely a consequence of increased permissiveness of intestinal CD4 T cells to HIV/SIV infection due to high steady state activation status and significant expression of HIV-1 co-receptors including CCR5 and CXCR4 and the gut-homing receptor α4β7 [9–20].

Early studies in SIV-infected rhesus macaques suggested that intestinal lamina propria (LP) CD4 T cell death occurred both directly via lysis of productively infected cells as well as indirectly via apoptotic death of bystander cells [3, 21, 22]. To study the mechanisms that drive HIV-mediated CD4 T cell death in intestinal tissues, we employed a human intestinal Lamina Propria Aggregate Culture (LPAC) model. We demonstrated that apoptosis was the primary death pathway in productively infected CD4 T cells and caspase-1-mediated T cell pyroptosis was the primary mechanism for bystander LP CD4 T cell death [23]. These observations were in agreement with Doitsh and colleagues who demonstrated in ex vivo cultures of tonsil tissues infected with X4-tropic HIV-1 that the majority of CD4 T cell death occurred in bystander cells as a result of abortive HIV infection, accumulation of incomplete HIV reverse transcripts and death by pyroptosis [24, 25].

Loss of protective gut Th17 and Th22 cells, along with disruption of epithelial barrier integrity (reviewed in [26]) leads to the translocation of luminal bacteria and bacterial products into the LP and subsequently into the systemic circulation [27, 28], termed microbial translocation. The process of microbial translocation occurs throughout all stages of HIV-1 infection and is considered to be a major contributor to chronic immune activation [27–29]. Moreover, despite decreases in levels of microbial translocation after initiation of ART, persistence of microbial translocation is associated with poor CD4 T cell recovery and non-AIDS morbidity and mortality [29–37]. In the setting of microbial translocation, mucosal as well as systemic immune cells are exposed to intestinal bacteria and their products. Increased levels of *E. coli* and lipopolysaccharide (LPS), a gram-negative bacterial cell wall component, were observed in the colonic LP within 28 days post SIV infection [38]. In other SIV studies, translocating bacteria enriched for Proteobacteria were observed in the mesenteric lymph nodes of chronically SIV infected rhesus macaques [39]. We reported that levels of both LPS and lipoteichoic acid (LTA), a gram-positive cell wall component, were increased in the colonic LP of untreated HIV-1-infected study participants with a greater fraction of LP myeloid dendritic cells (mDCs) and macrophages found in association with LPS than LTA [40].

A number of recent studies have detailed significant alterations in the fecal and intestinal mucosal microbiomes during treated and untreated HIV-1 infection and highlighted a critical role for dysbiosis in driving mucosal and systemic immune activation [41–48]. The mechanisms by which dysbiosis contributes to inflammation are unclear, but we hypothesize that increased translocation of more ‘pathogenic’ bacterial species during HIV infection, coupled with a decrease in more ‘protective’ microbiota^,^ leads to stimulation of mucosal and systemic immune cells. We showed that a Prevotella-rich, Firmicutes-poor dysbiosis in untreated, HIV-1 infected participants was associated with colonic mDC activation, mucosal and systemic T cell activation, and microbial translocation [41]. *Prevotella* species that increased in abundance in the colonic mucosa during untreated HIV-1 infection were associated with colonic mDC activation levels in vivo and directly activated mDCs in vitro [40]. Utilizing the LPAC model, we previously demonstrated that commensal *E. coli* activated bacteria-reactive intestinal T cells, augmented HIV-1 replication and infection of CD4 T cells [49, 50] and increased the death of productively infected cells through increased apoptosis in vitro [23]. However the impact of *Prevotella* species and other altered commensal bacterial species on mucosal infection and T cell depletion during HIV-1 infection remains unclear.

Here, we sought to better understand how different bacterial species, in particular those altered in the mucosa of HIV-1-infected individuals, may impact CD4 T cell infection and depletion using the LPAC model. Specifically, we studied a panel of representative HIV-altered mucosal bacteria (HAMB) that increased or decreased in relative abundance in the colonic mucosa of untreated, viremic HIV-1 infected individuals [40, 41]. We show that, although all HAMB increased HIV infection and depletion of LP CD4 T cells to some degree, gram-negative HAMB appeared to enhance infection and depletion to a greater extent than gram-positive HAMB. Furthermore, we provide evidence that the increased levels of CD4 T cell infection were likely a consequence of bacteria-induced enhancement of CCR5 expression on CD4 T cells through indirect mechanisms.

## Results

### HIV-altered mucosal bacteria (HAMB) species differentially increased productive HIV-1 infection and LP CD4 T cell depletion in vitro

**Fig. 1:**
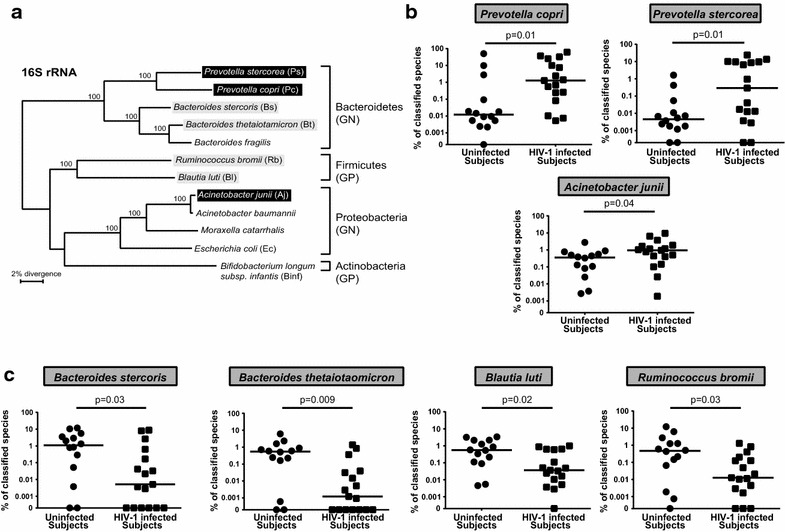
Abundances of HIV-altered mucosal bacteria (HAMB) species in HIV-infected and uninfected study participants. Bacterial taxa were identified in colon biopsies from 17 participants with chronic, untreated HIV-1 infection and 14 uninfected control participants using bacterial 16S ribosomal DNA sequencing. Of the 21 species that were significantly over (6) or under (15) represented in HIV-1-infected participants (Additional file 1: Table S1), 7 species that represented each of the 3 phyla (Bacteroides, Proteobacteria and Firmicutes) were evaluated in the LPAC model. **a** Phylogenetic tree illustrating taxonomic hierarchy for each HAMB species (*GN* gram-negative; *GP* gram-positive). **b**, **c** Plots showing individual relative abundance of each species that were (**b**) increased or (**c**) decreased in HIV-1 infected participants (*squares*) compared to uninfected participants (*circles*). Values are shown as a fraction of all classified species detected within each individual. *Lines* represent the median value. Statistical analysis was performed using the Mann–Whitney test

We recently identified 21 mucosa-associated bacterial species that were either increased or decreased in relative abundance in HIV-1 infected study participants compared to uninfected controls [40] (Additional file 1: Table S1). To investigate the potential impact of these HAMB [40] species on HIV-1 replication and CD4 T cell depletion using the LPAC model, we selected a panel of 7 HAMB species that represented each of the 3 major phyla (Bacteroidetes, Proteobacteria and Firmicutes) and that were identified in the majority of HIV-1 infected and uninfected study participants (Fig. 1). Gram-negative *P. copri*, *P. stercorea* (Bacteroidetes) and *A. junii* (Proteobacteria) were all significantly increased (Fig. 1b) in the colonic mucosa of HIV-1 infected participants and gram-negative *B. stercoris* and *B. thetaiotaomicron* (Bacteroidetes) were decreased (Fig. 1c). Gram-positive *B. luti* and *R. bromii* (Firmicutes) were also decreased in abundance in HIV-1 infected study participants (Fig. 1c).

**Fig. 2:**
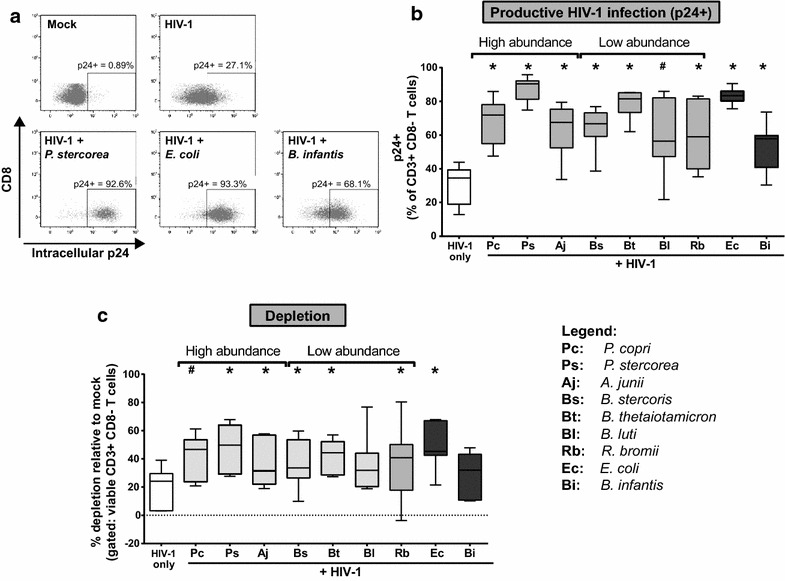
HAMB enhance HIV-1 infection and depletion of LP CD4 T cells. LPMC (n = 7) were spinoculated with CCR5-tropic HIV-1_BAL_ or mock control and exposed to High abundance or Low abundance HAMB species or to control bacteria (*E. coli*, *B. infantis*) (2.5 bacteria: 1 LPMC) for 4 days. LPMC were harvested and levels of productive infection and depletion of LP CD4 T cells determined. **a** Representative gating strategy illustrating intracellular p24 levels in viable CD4 T cells (gated as CD3+ CD8−) in presence or absence of bacteria with gates established on matched mock controls. **b** Percentages of intracellular p24-expressing (p24+) CD4 T cells in presence or absence of bacteria with background p24 (mock) values removed. **c** Levels of CD4 depletion in presence or absence of bacteria relative to depletion in matched mock conditions. Values are shown as ‘*box and whisker*’ with the *box* extending from the 25th to 75th percentile, the line in the box indicating the median value and the whiskers indicating maximum and minimum values.* White box* indicates no bacteria, *light gray boxes* indicate HAMB species and *dark gray boxes* indicate control bacteria. Statistical analysis was performed using the Wilcoxon matched–pairs signed rank test comparing frequencies of p24 + CD4 T cells or levels of depletion induced in response to bacteria to HIV-1 only. *p < 0.05, ^#^p = 0.08. Legend details the abbreviations used for each bacteria (x axis)

We first evaluated the impact of these HAMB species on productive HIV-1 infection levels in LP CD4 T cells in vitro using R5-tropic HIV_BaL_. Based on extensive data on the effect of enteric *E. coli* (gram-negative) on LP CD4 T cell infection, activation and depletion [23, 49], *E. coli* was included as a positive control. Lamina propria mononuclear cells (LPMCs) from 11 donors were infected with HIV_BaL_ in the presence or absence of *E. coli* and, at 4 days post infection (dpi), infection levels were evaluated by intracellular flow cytometry (Fig. 2a) and viable CD4 T cells counted. All but 4 donors exhibited >10 % CD4 T cell depletion with *E. coli*. These ‘depleters’ were used to compare the ability of different HAMB species to enhance LP CD4 T cell infection and depletion. In addition, the probiotic *Bifidobacterium longumsubspecies infantis* (*B. infantis*) was included in these studies. Of note, the abundances of *E. coli* or *B. infantis* were not significantly different in our cohort of untreated HIV-1 infected study participants compared to uninfected participants (p = 0.63 and p = 0.53 respectively; data not shown).

*E. coli* significantly enhanced HIV-1 infection in LP CD4 T cells as measured by intracellular p24 staining as expected [23, 49] (Fig. 2b). Interestingly, all HAMB species, irrespective of their abundance in the colonic mucosa of HIV-1 infected individuals significantly enhanced HIV-1 infection levels to varying degrees except *B. luti* (p = 0.08) (Fig. 2b). *B. infantis* significantly increased frequencies of p24+ CD4 T cells, although to levels generally lower than all other bacteria tested (Fig. 2b). Median frequencies of p24+ CD4 T cells induced in response to gram-negative bacteria were all higher than those induced in response to gram-positive bacteria. However, there were differences in the extent to which gram-negative bacteria influenced productive infection. Of the gram-negative HAMB species tested, *P. stercorea* induced the highest levels of productive infection, similar in magnitude to *E. coli.*

Next, we assessed the impact of HAMB on HIV-1 mediated CD4 T cell depletion (Fig. 2c). Nearly all HAMB species increased HIV-1-associated CD4 T cell depletion relative to HIV-1 infection without HAMB. *P. stercorea* induced the highest levels of depletion, similar to depletion levels induced in response to *E. coli*, whereas enhancement of CD4 T cell depletion by *P. copri* did not reach statistical significance (p = 0.08). Two species that induced lower levels of HIV-1 infection, *B. luti* and *B. infantis* (Fig. 2b), also did not significantly enhance CD4 T cell depletion (Fig. 2c).

To directly assess if a relationship existed between HIV-1 infection and depletion levels, p24+ frequency values or depletion values in response to each HAMB (n = 7) from each LPMC donor (n = 7) were pooled (n = 49 values) and correlation analyses performed. Productive HIV-1 infection induced in response to HAMB significantly correlated with depletion (r = 0.43, p = 0.002; Additional file 2: Figure S1). This result was consistent with our previous observation that frequencies of p24+ LP CD4 T cells predicted depletion and that depletion was dependent on productive HIV-1 infection [23].

### HAMB induced low levels of LP CD4 T cell activation and proliferation

**Fig. 3:**
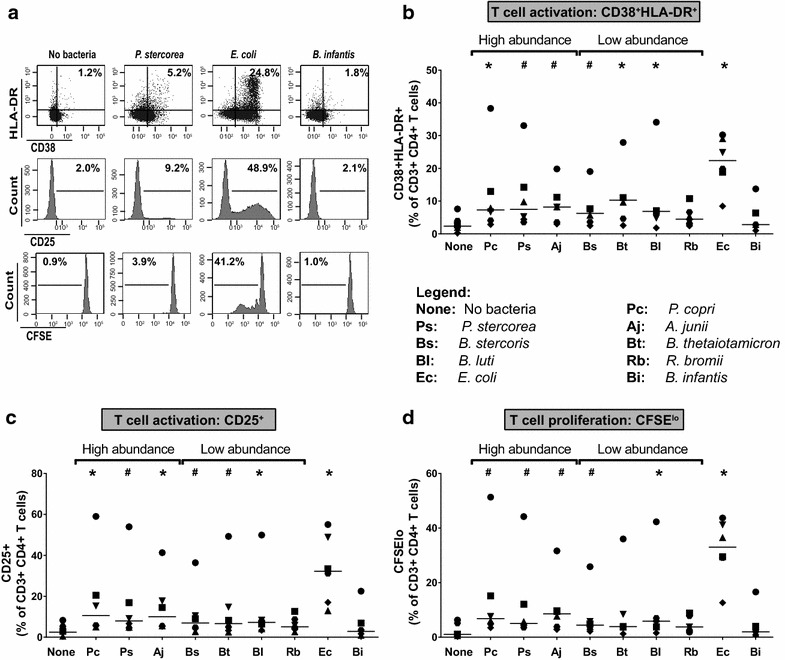
HAMB induce low levels of LP CD4 T cell activation and proliferation. LPMC (n = 6) were pre-labeled with CFSE and exposed to High abundance or Low abundance HAMB species or to control bacteria (*E. coli*, *B. infantis*) (2.5 bacteria: 1 LPMC) for 5 days. LPMC were harvested and frequencies of activated (CD38^+^HLA-DR^+^, CD25^+^) and proliferating (CFSE^lo^) LP CD4 T cells were evaluated using flow cytometry.** a** Representative gating strategy illustrating CD38^+^HLA-DR^+^, CD25^+^ and CFSE profiles of viable LP CD4 T cells in presence or absence of bacteria with gates established on media (CD25^+^, CFSE) or FMO controls (CD38/HLA-DR).** b** Percentages of LP CD4 T cells co-expressing CD38 and HLA-DR. FMO control values have been subtracted. **c** Percentages of CD4 LP T cells expressing CD25. **d** Percentages of CFSE^lo^ LP CD4 T cells. Values are shown as *symbols* representing each individual donor to highlight the 1 donor that exhibited unusually high responses to HAMB. *Line* indicates the median value. Statistical analysis was performed using the Wilcoxon matched–pairs signed rank test comparing percentages of activated or proliferating LP CD4 T cells induced in response to bacteria to no bacteria. *p < 0.05, ^#^p = 0.06. Legend details the abbreviations used for each bacteria (x axis)

We previously showed that the increased susceptibility of LP CD4 T cells to HIV-1 infection following exposure to *E. coli* was linked to increased CD4 T cell activation and proliferation [49]. We therefore evaluated bacteria-induced T cell activation, defined as LP CD4 T cells co-expressing CD38 and HLA-DR (CD38+ HLA-DR+) (Fig. 3a). All HAMB except *R. bromii* increased the percentages of CD38+ HLA-DR+ CD4 T cells to some degree. *P. copri*, *B.thetaiotaomicron* and *B. luti* significantly increased the percentage of CD38+ HLA-DR+ CD4 T cells compared to unstimulated cultures (Fig. 3b). *P. stercorea*, *A. junii* and *B. stercoris* also increased the fraction of CD4 T cells co-expressing CD38 and HLA-DR but did not reach statistical significance (p = 0.06) (Fig. 3b). Notably, the average fold increase in CD4 T cell activation induced in response to HAMB (*P. copri*: 5.4×; *P. stercorea*: 6.4×, *A. junii*: 5.2×; *B. stercoris*: 4.2×, *B.thetaiotaomicron*: 5.6×, *B. luti*: 4.0, *R. bromii*: 3.6×) were substantially lower than that measured in the presence of *E. coli* (14.4×).

We also evaluated the percentages of CD25-expressing CD4 T cells as an additional indicator of T cell activation (Fig. 3a). Similar to activation assessed as CD38 and HLA-DR co-expression, HAMB-induced increases in CD25 expression varied, but were considerably lower (average fold increase: *P. copri*: 7.0×; *P. stercorea*: 6.4×, *A. junii*: 6.7×; *B. stercoris*: 4.3×, *B.thetaiotaomicron*: 4.6×, *B. luti*: 4.7, *R. bromii*: 3.5×) than those observed in *E. coli*-exposed cultures (17.1×) (Fig. 3c).

We next assessed levels of CD4 T cell proliferation induced in response to bacteria (Fig. 3a). CD4 T cell proliferative responses to HAMB exposure were generally much lower than those observed following exposure to *E. coli* (Fig. 3d). Thus, relative to *E. coli*, the HAMB species induced significantly lower levels of CD4 T cell activation and proliferation.

### HAMB increased CCR5 expression levels on CD4 T cells

**Fig. 4:**
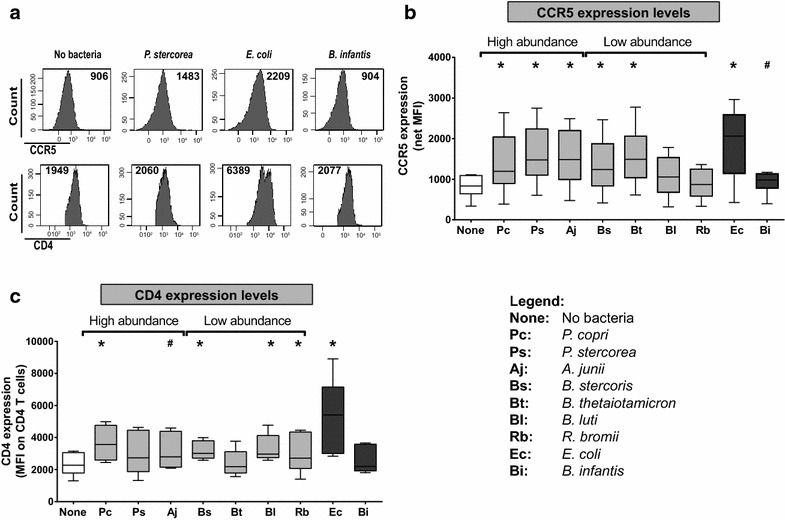
HAMB increase CCR5 expression on LP CD4 T cells. LPMC (n = 6) were exposed to High abundance or Low abundance HAMB species or to control bacteria (*E. coli*, *B. infantis*) (2.5 bacteria: 1 LPMC) for 5 days. LPMC were harvested and expression levels of HIV-1 co-receptors CCR5 and CD4 on LP CD4 T cells determined using flow cytometry. **a** Representative gating strategy illustrating CCR5 and CD4 expression on viable LP CD4 T cells in presence or absence of bacteria. **b** Expression levels (Mean fluorescence intensity; MFI) of CCR5 on CD4 LP T cells. Isotype control values have been subtracted (net MFI). **c** CD4 expression levels (MFI) on LP CD4 T cells. Values are shown as ‘*box and whisker*’ with the *box* extending from the 25th to 75th percentile, the *line* in the *box* indicating the median value and the whiskers indicating maximum and minimum values. *White box* indicates no bacteria, *light gray boxes* indicate HAMB species and *dark gray boxes* indicate control bacteria. Statistical analysis was performed using the Wilcoxon matched–pairs signed rank test comparing CCR5 or CD4 expression levels on LP CD4 T cells induced in response to bacteria to no bacteria. *p < 0.05, ^#^p = 0.06. Legend details the abbreviations used for each bacteria (x axis)

To further probe potential mechanisms driving HAMB-enhanced productive infection of CD4 T cells with a CCR5-tropic virus, we next evaluated the expression of the HIV-1 co-receptors CCR5 and CD4 on LP CD4 T cells following bacterial exposure (Fig. 4a). CCR5 expression levels were significantly increased following exposure to all gram-negative HAMB but not for gram-positive *B. luti* and *R. bromii* (Fig. 4b). On average, the fold increase in CCR5 expression on CD4 T cells in response to exposure to each gram-negative HAMB (*P. copri*: 1.7×; *P. stercorea*: 2.0×, *A. junii*: 1.9×; *B. stercoris*: 1.7×, *B.thetaiotaomicron*: 2.0×) was comparable to that induced by *E. coli* (2.3×). Only *P. copri*, *B. stercorea*, *B. luti* and *R. bromii* significantly increased CD4 expression levels on CD4 T cells and to levels generally lower than those measured on CD4 T cells in response to exposure to *E. coli* (Fig. 4c).

### Bacteria-induced increases in CCR5 expression may be mediated in part by the cell wall component LPS

**Fig. 5:**
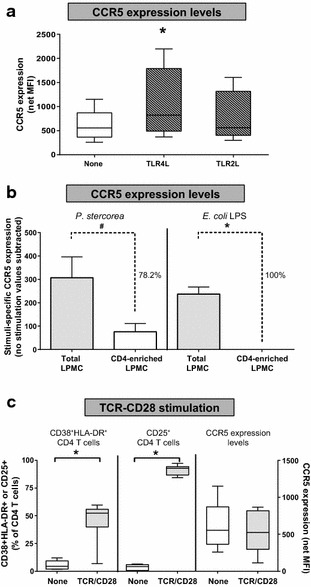
HAMB-mediated increased CCR5 expression is a result of indirect stimulation of LP CD4 T cells. **a** CCR5 expression in response to individual TLR ligands (TLRL) was measured by exposing LPMC (n = 6–7) to TLR4L (commensal *E. coli* LPS, 1 μg/ml) or TLR2L (*Bacillus subtilis* LTA, 1 μg/ml) or cultured without TLRLs (None) for 5 days. LPMC were harvested and CCR5 expression levels (Mean fluorescence intensity; MFI) on LP CD4 T cells evaluated. Isotype control values have been subtracted (net MFI) and values are illustrated as ‘*box and whisker*’ with the *box* extending from the 25th to 75th percentile, the *line* in the *box* indicating the median value and the whiskers indicating maximum and minimum values. Statistical analysis was performed using the Wilcoxon matched–pairs signed rank test, *p < 0.05. **b** Levels of CCR5 expression on LP CD4 T cells in response to stimulation of total LPMC or CD4-enriched LP cells with *P. stercorea* (2.5:1 LPMC/CD4 T cell) or *E. coli* LPS (1 μg/ml) for 24 h. Values shown are mean ± SEM of stimuli–specific CCR5 expression levels (net; no stimulation values subtracted; n = 3). Percent values indicate the average decrease in levels of CCR5 expression on CD4 T cells stimulated directly relative to expression levels on CD4 T cells from total LPMC stimulated cultures. Statistical analysis was performed using Paired t test, *p < 0.05, #p = 0.07. (C) LPMC (n = 6–7) were exposed to CD3/CD2/CD28 beads (1 bead: 2 LPMC) or cultured without exogenous stimuli (None) for 5 days. LPMC were harvested and frequencies of activated (CD38^+^HLA-DR^+^; CD25^+^) LP CD4 T cells and CCR5 expression levels determined. Values are shown with isotype (CCR5) or FMO control values removed and illustrated as ‘*box and whisker*’ plots. Statistical analysis was performed using the Wilcoxon matched–pairs signed rank test. *p < 0.05

To dissect potential mechanisms behind the upregulation of CCR5 expression in response to exposure to whole bacteria, we investigated CCR5 expression levels on CD4 T cells in response to exposure of LPMC to individual bacterial products highly expressed in the surface membrane of gram-negative (LPS) and gram-positive (LTA) bacteria. LPS significantly increased CCR5 expression whereas exposure to LTA did not (Fig. 5a). This lack of CCR5 induction was not related to LTA concentration as increasing doses of LTA (up to 50 μg/ml) failed to significantly induce CCR5 expression (data not shown).

To determine whether CCR5 upregulation by gram-negative bacteria occurred early in culture and thereby lead to enhanced HIV-1 infection of CD4 T cells, LPMC from 7 donors were exposed to *P. stercorea* or LPS and CCR5 expression levels evaluated after 24 h. At 24 h, significant increases in CCR5 expression on CD4 T cells were observed in response to both *P. stercorea* (median mean fluorescence intensity (MFI): 1723, range 592–3577; p = 0.016) and LPS (1407, 577–3062; p = 0.016) above no stimulation (1183, 463–2365).

### Bacteria-associated increases in CCR5 expression on LP CD4 T cells occurs indirectly and is not mediated through TCR signaling

We previously showed that commensal *E.coli*-associated enhancement of productive HIV-1 infection of LP CD4 T cells was dependent on the presence of LP mDC and was, in part, mediated through an MHC-Class II dependent mechanism [49]. Therefore, we next determined whether upregulation of CCR5 on T cells by *P. stercorea* and LPS was mediated directly via bacteria/T cell interaction or indirectly (potentially through bacteria/antigen presenting cell (APC) interactions). LP cells highly enriched for CD4 T cells (>92 % of viable CD45+ cells) were stimulated in the presence or absence of *P. stercorea* or LPS and levels of CCR5 expression on CD4 T cells compared to CCR5 expression levels on CD4 T cells induced in total LPMC cultures (Fig. 5b). In the presence of *P. stercorea*, levels of CCR5 expression on CD4 T cells were, on average, 78 % (±10.5 % SEM) lower in CD4 T cell-enriched cultures compared to CD4 T cells levels measured when total LPMC were exposed to *P. stercorea*. Exposure of CD4 T cell-enriched cultures to LPS completely failed to induce CCR5 expression (Fig. 5b).

To determine if direct TCR stimulation increased CCR5 expression on LP CD4 T cells, we exposed LPMC to beads that signal through CD3, CD2 and CD28. As expected, direct TCR-mediated signaling significantly increased expression of CD4 T cell activation markers CD38 and HLA-DR (Fig. 5c). However, levels of CCR5 on CD4 T cells were not increased after 5 days of stimulation of LPMC with CD3/CD2/CD28 beads compared to levels observed in unstimulated cultures (Fig. 5c).

## Discussion

HIV-1 infection is associated with major structural and immunological disruption of the GI tract due in part to high levels of HIV-1 replication and CD4 T cell depletion that occur in the earliest stages of infection. In recent years, a number of research groups including ours [40–48], have linked alterations in the intestinal microbiome, termed dysbiosis, in HIV-1 infected individuals to intestinal and systemic immune activation. We previously demonstrated that *E. coli*, an enteric bacterial species, augmented HIV-replication in LP CD4 T cells and drove apoptotic T cell death in vitro [23, 49]. However, our recent analyses revealed that *E. coli* abundance was not significantly different in colonic mucosa of untreated, viremic HIV-1 infected individuals compared to uninfected study participants. Instead, other bacterial species, particularly *Prevotella* species, were altered [40, 41]. Increased abundances of *Prevotella* in fecal and colonic tissue samples from untreated and treated HIV-1 infected individuals have also been reported by others [44, 45, 47, 51]. However, only one other group identified these down to the species level and in fecal rather than mucosal samples [44]. The study by Lozupone and colleagues showed HIV-associated increased fecal abundances of *P. copri* and *P. stercorea* and decreased abundances of *B. thetaiotaomicron* and *A. putredinis,* [44] similar to our observations in the colonic mucosa of untreated HIV-1-infected individuals. This current study was undertaken to further understand the potential mechanisms by which altered intestinal microbiota adherent to the colonic mucosa (HAMB), and therefore more likely to interact with intestinal immune cells, may contribute to HIV-1-associated mucosal pathogenesis. We utilized the LPAC model [23] to investigate the impact of HAMB species on LP CD4 T cell infection and depletion in vitro. We further used this ex vivo model system to probe potential microbiota-associated mechanisms that drive these responses.

Exposure of LP CD4 T cells to a panel of HAMB resulted in enhanced productive CCR5-tropic HIV-1 infection, similar to our previous results with *E.coli* [23, 49]. In particular, commensal *E. coli* strongly enhanced HIV-1 replication in LPMCs and this was linked to high levels of LP CD4 T cell activation and proliferation [49]. Surprisingly, the levels of CD4 T cell activation and proliferation induced in response to HAMB were substantially lower than those observed in response to *E. coli*. This apparent disconnect between HAMB-associated increased T cell activation and productive HIV-1 infection was most apparent for *P. stercorea*, which enhanced productive HIV-1infection to similar levels as induced by *E. coli*. These observations suggest that mechanisms other than increased T cell activation underlie the observed HAMB-mediated enhancement of HIV-1 infection.

High expression levels of CCR5, an HIV co-receptor, on LP CD4 T cells has also been implicated in their natural permissivity and susceptibility to HIV infection [10, 16, 18, 19, 52]. We theorized that modulation of CCR5 expression might be one mechanism responsible for bacteria-induced increased T cell infectivity with R5-tropic virus. Consistent with this theory, CCR5 was significantly up-regulated on LP CD4 T cells early after exposure to HAMB. In fact, the levels of CCR5 induced in response to certain gram-negative HAMB (i.e. *P. stercorea*) were approaching the high levels induced in response to *E. coli*. Notably, increased CCR5 expression on blood and lung CD4 T cells in patients with tuberculosis with or without HIV-1 infection was proposed as a mechanism for the increased HIV viremia and hastened course of HIV progression associated with tuberculosis co-infection [53–55]. Exposure of whole blood to mycobacterial lipoarabinomannan in vitro increased CCR5 expression on blood CD4 T cells, suggesting the increased levels of CCR5 expression in tuberculosis patients was mediated, at least in part, by cell wall components of *Mycobacterium tuberculosis* [53]. Moreover, the oral pathogen *Porphyromonas gingivalis* increased CCR5 expression on oral keratinocytes in vitro, and may play a role in the selection of R5-tropic HIV-1 in the oral mucosa [56, 57]. We now provide the first evidence that commensal enteric bacteria are capable of augmenting CCR5 expression on intestinal LP CD4 T cells. We hypothesize that, in the context of dysbiosis and microbial translocation, this process further enhances productive infection by R5-tropic HIV-1 and contributes to the disruption of intestinal T cell homeostasis in HIV-1 infected individuals.

Regardless of taxonomic grouping and the relative levels of abundance in vivo, HAMB species enhanced HIV-1 infection in LP CD4 T cells. However, gram-negative bacteria increased productive infection to a greater degree than gram-positive bacteria. Potentially explaining this observation, exposure of LPMCs to the gram-negative bacterial cell wall component LPS enhanced CCR5 expression whereas exposure to LTA, a constituent of cell walls of gram-positive bacteria did not. This suggests that LPS may in part explain why gram-negative bacteria were particularly efficient at enhancing HIV-1 infection of LP CD4 T cells. Interestingly, all high abundance HAMB species were gram negative. Thus, in the context of microbial translocation, there would be a greater tendency for LP immune cells to be exposed to these gram-negative bacteria and their components. This implies a greater contribution of high abundance, gram-negative HAMB to HIV-associated LP immune dysfunction.

LPMCs are composed not only of effector memory CD4 T cells, but also of additional immune cells such as mDCs that direct immunogenic and tolerogenic intestinal immune responses [58] and play a role in viral dissemination [59]. In this study, the effects of both whole bacteria (*P. stercorea*) and LPS on CCR5 upregulation did not appear to result from direct stimulation of LP CD4 T cells. We previously showed a requirement for LP mDC in the activation and expansion of bacteria-reactive T cells as well as in the bacteria-associated enhancement of LP CD4 T cell infection mediated, in part, through MHC Class II-restricted antigen presentation [49, 50]. Our observations that direct T cell receptor activation in conjunction with CD28 co-stimulation of LP CD4 T cells did not result in CCR5 upregulation suggests that TCR-mediated activation by mDCs presenting bacterial antigens is unlikely to be solely sufficient to increase CCR5. Thus, our results suggest that bacterial antigens may prompt DCs, or other immune cells, to produce immune factors that then promote CCR5 upregulation on LP CD4 T cells. The mediators and signaling pathways involved in bacteria-associated CCR5 upregulation on LP CD4 T cells remain to be determined.

Although these studies begin to probe potential mechanisms by which an altered microbiome may drive HIV-associated mucosal pathogenesis, a limitation to the in vitro model is that it does not reflect the complexity of the microbiome in vivo, either in context of reflecting the microbial community as a whole or the collective impact that metabolites and other factors produced by bacteria may have on intestinal T cell infection and depletion in vivo.

## Conclusions

The present study provides the first evidence that commensal bacteria that are altered in the colonic mucosa of untreated, viremic HIV-1 infected individuals enhance both productive infection and depletion of LP CD4 T cells by CCR5-tropic HIV-1 ex vivo. Although all HAMB increased HIV-1 infection and depletion of LP CD4 T cells to some degree, gram-negative HAMB appeared to enhance infection and depletion greater than gram-positive HAMB. Somewhat unexpectedly, high abundance HAMB that induced significantly high levels of productive infection and CD4 T cell death similar to *E. coli* facilitated only minimal increased LP T cell activation. However, these same bacteria induced substantial increases in CCR5 expression on LP CD4 T cells. These observations highlight that bacteria may utilize different mechanisms to contribute to HIV-associated pathogenesis. Increases in CCR5 expression by HAMB were mediated by indirect stimulation of LP CD4 T cells and likely mediated in part through LPS signaling. Recognizing how dysbiosis impacts HIV-1 infection and death of intestinal CD4 T cells and determining the potential mechanisms that drive these bacteria-associated T cell responses will provide a better understanding of HIV-1-associated mucosal pathogenesis and lead to novel treatment approaches that target the microbiome and/or its downstream effects on the intestinal immune system.

## Methods

### Determination of colonic mucosa-associated bacterial species

Seventeen HIV-1 infected individuals and 14 HIV-1 seronegative (uninfected) controls who met the entry criteria were enrolled in this cross-sectional study at the University of Colorado Anschutz Medical Campus. Study design including study entry and exclusion criteria are extensively detailed in previous publications [40, 41]. Efforts were made to enroll HIV-1 seronegative study participants who were matched for age and sex to the HIV-1 infected participants. HIV-1 infected participants were ART-treatment naïve or had not been on treatment for more than 7 days in the preceding 6 months. The clinical details for study participants are provided in Additional file 3: Table S2. All study participants voluntarily gave written, informed consent. This study was approved by the Colorado Multiple Institutional Review Board (COMIRB) at the Anschutz Medical Campus.

Laboratory and analytic methods used to profile the intestinal microbiomes of study participants were described previously [40, 41]. Briefly, species-level taxonomic classification of 16S rRNA sequence datasets was obtained via BLAST [60] of subject sequences against a database built from Silva [61] bacterial sequences marked as type strains, cultivars, or genomes. A species name was assigned when a sequence overlapped the Silva database sequence by at least 95 % sequence length with at least 99 % sequence identity and the taxonomy of the database hit matched the taxonomy returned by SINA [62] as determined previously [41]. Most sequences were classified assignable to a species (median 97.4 %, interquartile range 94.6–98.6 %).

### Human intestinal Lamina Propria Aggregate Culture (LPAC) model

Human jejunum tissue samples (n = 16) were obtained from patients undergoing elective abdominal surgery, represent otherwise discarded tissue and were considered macroscopically normal as previously described [23, 40, 49, 50, 63]. Those with a history of inflammatory bowel disease, recent chemotherapy or radiation or immunosuppressive drugs were excluded from the study. All patients undergoing surgery signed a release to allow the unrestricted use of discarded tissues for research purposes, and all protected patient information was de-identified to the laboratory investigators. This research was reviewed by the Colorado Multiple Institutional Review Board (COMIRB) at the University of Colorado Anschutz Medical Campus and was granted exempt research status. LPMC were isolated from tissue samples and released LPMC were cryopreserved and stored in liquid nitrogen as detailed elsewhere [23, 40, 49, 50, 63].

### Preparation of commensal bacterial stocks

Expansion of *P. copri* (DSM No. 18205, DSMZ, Braunschweig, Germany), *P. stercorea* (DSM No. 18206), *B. stercoris* (ATCC 43183, ATCC Manassas, VA, USA), *B. thetaiotaomicron* (ATCC 29741), *B. luti* (DSM No. 14534), *R. bromii* (ATCC# 27255) and *Bifidobacterium longum subsp infantis* (ATCC 15697) was performed at 37 °C under anaerobic conditions achieved by using a BD GasPak EZ Anaerobe Pouch System (BD Diagnostics, Franklin Lakes, NJ, USA) per manufacturer’s protocols with slight modifications. Specifically, *P. copri* was expanded by culturing on Brucella plates (BD Diagnostics) for 5–7 days; *P. stercorea* was expanded in liquid chopped meat broth (Hardy Diagnostics, Santa Maria, CA, USA) supplemented with 1 % Trace Minerals (ATCC), 1 % Vitamin Supplements (ATCC), 0.05 % Tween80, 29.7 mM acetic acid, 8.1 mM propionic acid and 4.4 mM butyric acid (all Sigma-Aldrich) for 5–7 days; *B. stercoris*, *B. thetaiotaomicron*, *B. luti*, *R. bromii* and *Bifidobacterium longum subsp infantis* were expanded in liquid chopped meat broth (BD Biosciences; Hardy Diagnostics) for 1–2 days with the exception of *Bifidobacterium longum subsp infantis* which was expanded for 2–3 days. Expansion of *A. junii* (ATCC 17908) and *E. coli* (ATCC 25922) was performed under aerobic conditions at 26 and 37 °C respectively. *A. junii* was expanded using Nutrient Agar plates (Edge Biologicals, Memphis, TN, USA) and *E. coli* expanded in LB broth (Sigma-Aldrich) for 1–2 days. All bacteria were stored long-term at −80 °C in 10 % glycerol. To prepare working stocks, bacteria were expanded from long-term stocks as described above except for *B. stercoris*, *B. thetaiotaomicron* and *Bifidobacterium longum subsp infantis* which were grown on Brucella plates and *E. coli* which was grown on LB Agar plates (Teknova, Hollister, CA, USA). Bacteria were suspended in DPBS and stored at −80 °C in single-use aliquots. Bacterial enumeration was performed as previously detailed [40].

### Preparation of CCR5-tropic HIV-1_Ba-L_ stocks

Preparation of HIV-1 viral stocks was performed as previously detailed [23]. Briefly, MOLT4-CCR5 cells (AIDS Research and Reference Reagent Program; ARRP Catalog# 510) were infected with R5-tropic HIV-1_Ba-L_ strain (ARRP #4984) or mock-infected in the presence of polybrene (Santa Cruz Biotechnology, Dallas, TX, USA). Additional MOLT4-CCR5 cells were provided 4 days post infection (dpi) and culture supernatants collected 9dpi. Concentrated viral stocks were obtained by ultracentrifugation at 141,000×*g* for 2 h. Concentrations of p24 in the supernatant was determined by HIV Gag p24 ELISA (Perkin Elmer, Walthman, MA, USA). Virus and mock stocks were frozen in single use aliquots at −80 °C.

### LPMC infection assay

The LPMC infection assay that has been previously detailed [23] was used with slight modifications to account for a 96-well plate format. LPMCs were thawed using a standard protocol as detailed [23] and resuspended in RPMI + 10 % human AB serum (Gemini Bioproducts, West Sacramento, CA, USA) + 1 % penicillin/streptomycin/glutamin (Life Technologies, Grand Island, NY, USA) (cRPMI) + 500 µg/ml Zosyn (piperacillin/tazobactium; Wyeth, Madison, NY, USA) (cRPMI). HIV-1_Ba-L_ (10 ng p24 per 1 × 10^6^ LPMCs) was used to infect primary LPMCs at 2.5 × 10^6^ LPMCs/ml. LPMCs were mock infected in parallel. Infection by spinoculation was performed at 1500×*g* for 2 h at room temperature. After 2 h supernatant containing the free virus was discarded and the LPMCs were washed with cRPMI. LPMCs were resuspended at 1 × 10^6^ LPMCs/ml in cRPMI and plated into triplicate wells of a 96-well V-bottom culture dish and live bacteria added where appropriate. LPMCs were cultured for 4 days at 37 °C, 5 % CO_2_ and 95 % humidity.

### LPMC stimulation with commensal bacteria, TLR ligands and T cell activation beads

Whole bacteria was added to cell cultures at 2.5 bacteria: 1 LPMC as previously described [40]. Commensal *E. coli* LPS (InvivoGen, San Diego, CA, USA) and *B. subtilis* LTA (InvivoGen) were added at 1 μg/ml. For TLR ligand dose titration assays, LPS and LTA were also added at 5 and 50 μg/ml. T cell receptor (TCR)-mediated T cell activation was performed using CD3/CD2/CD28 beads (T cell Activation/Expansion Kit, Miltenyi Biotec, Auburn, CA, USA) at a ratio of 1 bead to 2 LPMC.

### Measurement of T cell proliferation, activation and CCR5 expression

To measure levels of T cell proliferation and activation in response to the panel of 7 HAMB, LPMC were pre-labelled with 1 μM CellTrace CFSE (Invitrogen, Grand Island, NY, USA) per manufacturer’s instructions. CFSE-labeled cells were mock-infected by spinoculation and plated in triplicate in 96-well plates as detailed above. LPMCs were cultured with or without bacteria for 5 days at 37 °C, 5 % CO_2_ and 95 % humidity. Total LPMCs were collected and CD4 T cell proliferation, levels of T cell activation and expression levels of CCR5 were determined by multi-color flow cytometry.

To determine T cell activation and CCR5 expression levels in response to TLR ligands, TCR-mediated signaling and *P. stercorea*, LPMC were cultured in cRPMI in a 48well plate for 24 h or 5 days at 37 °C, 5 % CO_2_ and 95 % humidity in the presence or absence of *E. coli* LPS, *B. subtilis* LTA, CD3/CD2/CD28 beads or bacteria and levels of T cell activation and expression levels of CCR5 determined by multi-color flow cytometry.

### Enrichment of LP CD4 T cells

LPMC were highly enriched for CD4 T cells using column-based immunomagnetic selection and the EasySep magnet (Stemcell Technologies, Vancouver, BA, Canada). Firstly, LPMC were stained with biotinylated CD11c (eBioscience, San Diego, CA, USA) and control LPMC (total LPMC) resuspended in buffer only. FcR blocking reagent (Miltenyi Biotec) was added to all conditions. Biotinylated CD11c-bound cells were removed using the EasySep Biotin Selection Kit (Stemcell Technologies) following the manufacturers protocol. Next, CD11c-depleted LPMC were enriched for CD4 LP T cells using the EasySep Human CD4+ T cell isolation kit (Stemcell Technologies) per manufacturers’ protocol. Control LPMC were incubated with buffer in place of the CD4+ T cell Enrichment Cocktail. All other steps in the protocol were the same as those followed to enrich for LP CD4 T cells. Multi-color flow cytometry was used to evaluate CD4 T cell frequencies as well as frequencies of non-CD4 T cells (CD8 T cells, γδ T cells, B cells, mDC) in both CD4-enriched LPMC and in total LPMC. On average, CD4 T cells were enriched to 92.9 % (of viable, CD45+ cells; n = 3). CD4-enriched LPMC or total LPMC were cultured for 24 h at 37 °C, 5 % CO_2_ and 95 % humidity in the presence or absence of P. stercorea or *E. coli* LPS and levels of expression of CCR5 on CD4 T cells determined by multi-color flow cytometry.

### Surface and intracellular flow cytometry staining assays

All antibodies and dyes are listed in Additional file 4: Table S3. Standard flow cytometry staining protocols to determine expression of surface markers (LP cell identification, proliferation, activation, HIV-1 co-receptor expression) and intracellular expression of HIV-1 core antigen (p24) were followed as previously detailed [23, 49].

### Flow cytometry acquisition and analysis

To measure productive infection (p24), flow cytometry data was acquired on a Gallios 561 flow cytometer (Beckman Coulter) using the Hypercyte 96-well plate reader and analyzed using Kaluza software version 1.2 (Beckman Coulter). All other flow cytometry data was acquired on an LSRII flow cytometer (BD Biosciences) and analyzed using BD FACS DIVA version 6.1.3 as previously detailed [49].

To identify LP CD4 T cells, a total lymphocyte gate based on forward and side scatter properties was determined in viable (aqua dye^−^) cells and doublets excluded based on forward-scatter-height versus forward-scatter-width properties. HIV-1 down-regulates CD4 expression [64], therefore frequencies of p24-expressing LP CD4 T cells were determined by gating CD4 T cells as CD3+ CD8− T cells as previously detailed [23, 49]. For all other assays, CD4 T cells were identified as CD3+ CD4+ CD8− T cells. Proliferating CD4 T cells were enumerated as the percentage of CFSE^lo^ CD4 T cells with the CFSE gate established on the unstimulated condition. T cell activation was determined by evaluating the percentage of CD4 T cells that co-expressed CD38 and HLA-DR as well as the percentage of cells that expressed CD25. Expression of CCR5 was evaluated as the mean fluorescence intensity (MFI) on CD4 T cells. Expression levels of CD4 on CD4+ T cells were also assessed as MFI. In all cases except expression levels of CD4 on CD4 T cells, isotype or FMO control values were subtracted and data displayed as ‘net’ values.

To quantify depletion, the difference in the number of viable T cells in HIV-1-infected cultures compared to the number of viable T cells in matched mock-infected cultures was reported as the percentage of viable T cells in mock-infected control wells [23]. For example, if 100,000 viable T cells were measured in an HIV-infected well and 500,000 viable T cells measured in mock-infected cultures, the percent depletion would be reported as 80 %. The number of absolute viable T cells in each condition was calculated as previously detailed with some modifications [23]. Specifically, LPMC were collected at the completion of the culture period and counted using a TC20 Automated Cell Counter (Bio-Rad Laboratories, Hercules, CA, USA) with viable cells calculated based on Trypan Blue (Hyclone Laboratories, Logan, UT, USA) exclusion. The viable cell count was then multiplied by the percentage of viable CD3+ CD8− T cells measured by flow cytometry to determine the absolute number of viable cells in each well. All conditions were run in triplicate wells with the average depletion for the 3 wells reported.

### Statistical analysis

Statistical analysis and graphing were performed using GraphPad Prism version 6 for Windows (GraphPad Software, San Diego, CA, USA). Non-parametric tests were performed to determine differences between groups of matched paired data using Wilcoxon’s matched-pairs signed-rank test and Spearman test to determine correlations between variables. A paired t test was used to evaluated differences between groups in the CD4− enriched LPMC assays due to small sample size (n = 3).

## Additional files

10.1186/s12977-016-0237-1 Abundances of species in colonic mucosal tissue which are statistically different in HIV-infected subjects compared to uninfected subjects.
10.1186/s12977-016-0237-1 Levels of productive HIV-1 infection induced in response to HAMB correlate with levels of CD4 T cell depletion. LPMC (n=7) were spinoculated with CCR5-tropic HIV-1BAL or mock control and exposed to a panel of HIV-altered mucosal bacteria (HAMB; n=7) for 4 days. LPMC were harvested and levels of productive infection and depletion of LP CD4 T cells evaluated. To determine the association between induction of production infection and depletion in response to HAMB, values for each HAMB (n=7) from each donor (n=7) were pooled (n=49). Statistical analysis was performed using the Spearman test.
10.1186/s12977-016-0237-1 Clinical Study Participant Characteristics.
10.1186/s12977-016-0237-1 Antibodies and dyes used in multi-color flow cytometry protocols.

## Abbreviations

HIV: human immunodeficiency virus
SIV: simian immunodeficiency virus
ART: anti-retroviral therapy
GI: gastro-intestinal
LP: lamina propria
LPAC: Lamina Propria Aggregate Culture
LPS: lipopolysaccharide
LTA: lipoteichoic acid
mDCs: myeloid dendritic cells
HAMB: HIV-altered mucosal bacteria
LPMC: lamina propria mononuclear cells
MFI: mean fluorescence intensity
