# Supplementary material for: Enhancement of HIV-1 infection and intestinal CD4+ T cell depletion ex vivo by gut microbes altered during chronic HIV-1 infection
Source: Retrovirology. 2016 Jan 14;13:5. doi: 10.1186/s12977-016-0237-1 (PMC4712466; doi:10.1186/s12977-016-0237-1)
Supplement: Supplementary file 1 — 10.1186/s12977-016-0237-1 Abundances of species in colonic mucosal tissue which are statistically different in HIV-infected subjects compared to uninfected subjects. [file 12977_2016_237_MOESM1_ESM.docx]

**Additional File 1 Table S1. Abundances of species in colonic mucosal tissue which are statistically different in HIV-infected subjects compared to uninfected subjects.**

|  | Uninfected subjects (n=14) | | HIV-infected subjects (n=17) | | P value |
| --- | --- | --- | --- | --- | --- |
| Species | **Prevalence** | **% of classified species** | **Prevalence** | **% of classified species** |  |
| Bacteroidetes phylum |  |  |  |  |  |
| *Prevotella copri* | 13/14 | 0.0120 (0-49.99) | 17/17 | 1.266 (0.005-61.49) | **↑**^0.02^ |
| *Prevotella stercorea* | 12/14 | 0.0045 (0-1.638) | 15/17 | 0.2901 (0-24.04) | **↑**^0.01^ |
| *Prevotella oris* | 5/14 | 0 (0-14.23) | 12/17 | 0.0179 (0-3.982) | **↑**^0.01^ |
| *Bacteroides stercoris* | 12/14 | 1.082 (0-11.74) | 11/17 | 0.0051 (0-8.759) | ↓^0.03^ |
| *Bacteroides thetaiotaomicron* | 12/14 | 0.5477 (0-6.067) | 10/17 | 0.0012 (0-1.378) | ↓^0.009^ |
| *Bacteroides dorei* | 12/14 | 0.0622 (0-5.764) | 11/17 | 0.0016 (0-0.0743) | ↓^0.001^ |
| *Bacteroides acidifaciens* | 12/14 | 0.0082 (0-0.1599) | 10/17 | 0.0011 (0-0.0103) | ↓^0.02^ |
| *Alistipes putredinis* | 13/14 | 0.248 (0-1.512) | 8/17 | 0 (0-2.269) | ↓^0.03^ |
| *Barnesiella intestinihominis* | 11/14 | 0.0046 (0-2.582) | 9/17 | 0.0006 (0-1.251) | ↓^0.05^ |
| Firmicutes phylum |  |  |  |  |  |
| *Blautia luti* | 14/14 | 0.5504 (0.005-3.350) | 16/17 | 0.0359 (0-0.9864) | ↓^0.02^ |
| *Blautia glucerasei* | 12/14 | 0.4483 (0-1.872) | 14/17 | 0.0162 (0-0.9048) | ↓^0.03^ |
| *Blautia schinkii* | 11/14 | 0.0169 (0-2.851) | 9/17 | 0.0009 (0-0.0157) | ↓^0.005^ |
| *Blautia producta* | 7/14 | 0.0004 (0-0.0054) | 3/17 | 0 (0-0.0042) | ↓^0.04^ |
| *Rumminococcus bromii* | 13/14 | 0.4699 (0-12.13) | 14/17 | 0.0127 (0-1.287) | ↓^0.03^ |
| *Ruminococcus gnavus* | 10/14 | 0.0117 (0-0.6053) | 5/17 | 0 (0-0.0130) | ↓^0.005^ |
| *Clostridium saccharogumia* | 13/14 | 0.2914 (0-0.9406) | 11/17 | 0.0029 (0-1.842) | ↓^0.03^ |
| *Clostridium xylanovorans* | 10/14 | 0.0027 (0-2.195) | 6/17 | 0 (0-0.01845) | ↓^0.04^ |
| *Acidaminococcus intestini* | 8/14 | 0.0008 (0-6.514) | 16/17 | 0.0309 (0-3.781) | **↑**^0.03^ |
| *Bacteroides cellulosolvens* | 10/14 | 0.0161 (0-1.078) | 6/17 | 0 (0-0.1632) | ↓^0.01^ |
| Proteobacteria phylum |  |  |  |  |  |
| *Acinetobacter junii* | 14/14 | 0.3587 (0.0027-2.760) | 17/17 | 0.9451 (0.002-9.515) | **↑**^0.04^ |
| *Schlegelella thermodepolymerans* | 2/14 | 0 (0-0.0123) | 11/17 | 0.0021 (0-0.4952) | **↑**^0.003^ |

Values are shown as median, range. In each genus, species are ordered in based on highest abundance. Prevalence indicates the number of subjects in which each species was detected. *Arrows indicate an increase (↑) or decrease (↓) abundance in HIV-infected subjects compared to uninfected subjects. Statistical analysis was performed using Mann-Whitney test.
