# Supplementary material for: Enhancement of HIV-1 infection and intestinal CD4+ T cell depletion ex vivo by gut microbes altered during chronic HIV-1 infection
Source: Retrovirology. 2016 Jan 14;13:5. doi: 10.1186/s12977-016-0237-1 (PMC4712466; doi:10.1186/s12977-016-0237-1)
Supplement: Supplementary file 2 — 10.1186/s12977-016-0237-1 Levels of productive HIV-1 infection induced in response to HAMB correlate with levels of CD4 T cell depletion. LPMC (n=7) were spinoculated with CCR5-tropic HIV-1BAL or mock control and exposed to a panel of HIV-altered mucosal bacteria (HAMB; n=7) for 4 days. LPMC were harvested and levels of productive infection and depletion of LP CD4 T cells evaluated. To determine the association between induction of production infection and depletion in response to HAMB, values for each HAMB (n=7) from each donor (n=7) were pooled (n=49). Statistical analysis was performed using the Spearman test. [file 12977_2016_237_MOESM2_ESM.pdf]

Correlation between HAMB-induced productive HIV-1 infection and depletion in LP CD4 T cells

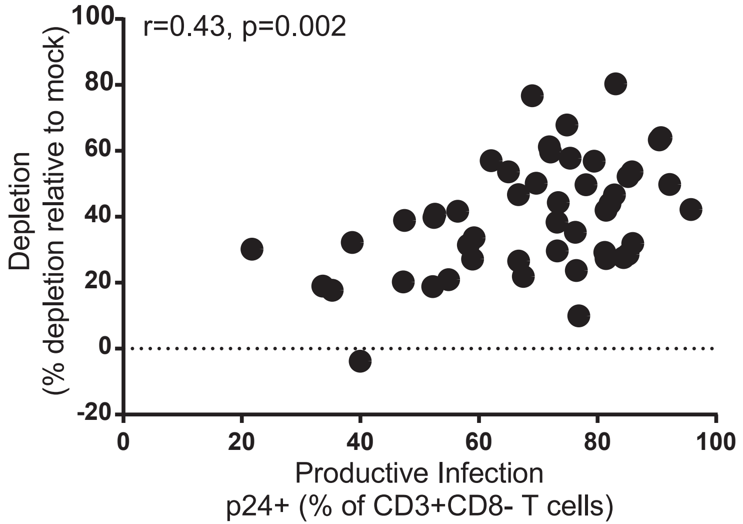

Additional File 2 Figure S1. Levels of productive HIV-1 infection induced in response to HAMB correlate with levels of CD4 T cell depletion. LPMC (n=7) were spinoculated with CCR5-tropic HIV-1_{BAL} or mock control and exposed to a panel of HIV-altered mucosal bacteria (HAMB; n=7) for 4 days. LPMC were harvested and levels of productive infection and depletion of LP CD4 T cells evaluated. To determine the association between induction of production infection and depletion in response to HAMB, values for each HAMB (n=7) from each donor (n=7) were pooled (n=49). Statistical analysis was performed using the Spearman test.
