# Supplementary material for: Enhancement of HIV-1 infection and intestinal CD4+ T cell depletion ex vivo by gut microbes altered during chronic HIV-1 infection
Source: Retrovirology. 2016 Jan 14;13:5. doi: 10.1186/s12977-016-0237-1 (PMC4712466; doi:10.1186/s12977-016-0237-1)
Supplement: Supplementary file 3 — 10.1186/s12977-016-0237-1 Clinical Study Participant Characteristics. [file 12977_2016_237_MOESM3_ESM.docx]

**Additional File 3 Table S2. Clinical Study Participant Characteristics.**

|  | Uninfected  subjects | HIV-1 infected  subjects |
| --- | --- | --- |
| Number of subjects | 14 | 17 |
| Age (yrs) | 31 (23-54) | 34 (25-58) |
| Male/Female Ratio | 9/5 | 12/5 |
| CD4 count (cells/μl) | 724 (468-1071) | 429 (238-782)* |
| Plasma Viral Load (HIV-1 RNA copies/ml) | - | 43200 (2880 – 196000) |
| Years since first HIV-1 seropositive test | - | 5.0 (0.25-15) |
| Body Mass Index (kg/m^2^) | 25.3 (18.5-32.3)^#^ | 26 (17.4-34.7) |
| Ethnicity: |  |  |
| Non-Hispanic | 11 (78.6%) | 16 (94.1%) |
| Hispanic | 3 (21.4%) | 1 (5.9%) |
| Race: |  |  |
| White/Caucasian | 10 (71.4%) | 12 (70.6%) |
| Black/African American | 2 (14.3%) | 4 (23.5%) |
| Asian | 2 (14.3%) | 1 (5.9%) |

Values are shown as median (range) except for Ethnicity and Race which are shown as the number and percentage of each cohort. Statistical analysis was performed using Mann-Whitney test for comparisons between uninfected and HIV-1 infected subjects and the Fisher Exact test or Chi-square test for comparison of categorical data. *p=0.0001. ^#^One subject had no weight or height values recorded at time of study therefore n=13.
