# Supplementary material for: Enhancement of HIV-1 infection and intestinal CD4+ T cell depletion ex vivo by gut microbes altered during chronic HIV-1 infection
Source: Retrovirology. 2016 Jan 14;13:5. doi: 10.1186/s12977-016-0237-1 (PMC4712466; doi:10.1186/s12977-016-0237-1)
Supplement: Supplementary file 4 — 10.1186/s12977-016-0237-1 Antibodies and dyes used in multi-color flow cytometry protocols. [file 12977_2016_237_MOESM4_ESM.docx]

**Additional File 4 Table S3. Antibodies and dyes used in multi-color flow cytometry protocols.**

|  | **Clone** | **Company** | **Location** |
| --- | --- | --- | --- |
| **Viable, mononuclear cell identification** | | | |
| Zombie Aqua Live/Dead Viability dye |  | Biolegend | San Diego, CA |
|  | |  |  |
| **Lamina Propria (LP) T cell infection** | |  |  |
| PerCP-Cy5.5 CD3 | OKT3 | Tonbo Biosciences | San Diego, CA |
| APC CD8 | RPA-T8 | Tonbo Biosciences |  |
| PE HIV-1 Core Antigen (p24) | KC57 | Beckman Coulter | Brea, CA |
|  |  |  |  |
| **LP T cell activation, CCR5 expression and proliferation** | | | |
| PerCP-Cy5.5 CD3 | OKT3 | Tonbo Biosciences |  |
| APC CD8 | RPA-T8 | Tonbo Biosciences |  |
| eFluor^450^ CD4 | RPA-T4 | eBioscience | San Diego, CA |
| AF700 CD38 | HIT2 | eBioscience |  |
| AF700 mouse IgG1 isotype control | P3.6.2.8.1 | eBioscience |  |
| APC-Cy7 HLA-DR | L243 | Biolegend |  |
| PE CD25 | M-A251 | BD Biosciences | San Jose, CA |
| PE-Cy7 CCR5 | 2D7 | BD Biosciences |  |
| PE-Cy7 mouse IgG2a isotype control | G155-178 | BD Biosciences |  |
| CFSE |  | Invitrogen | Carlsbad, CA |
| *In some assays, LP CD4 T cells were identified with the following antibodies:* | | | |
| PE Texas Red (ECD) CD3 | UCHT1 | Beckman Coulter |  |
| violetFluor^450^ CD4 | RPA-T4 | Tonbo Biosciences |  |
| PerCP-Cy5.5 CD8 | RPA-T8 | Tonbo Biosciences |  |
|  |  |  |  |
| **LP CD4 T cell enrichment** |  |  |  |
| PerCp-Cy5.5 CD45 | 2D1 | eBioscience |  |
| APC-Cy7 HLA-DR | L243 | Biolegend |  |
| AF700 CD11c | B-ly6 | BD Biosciences |  |
| APC BDCA-1 (CD1c) | AD5-8E7 | Miltenyi Biotec | Auburn, CA |
| APC mouse IgG2a isotype control | S43.10 | Miltenyi Biotec |  |
| PE Texas Red (ECD) CD3 | UCHT1 | Beckman Coulter |  |
| PE γδ TCR | 11F2 | BD Biosciences |  |
| PE-Cy7 CD8 | RPA-T8 | Tonbo Biosciences |  |
| violetFluor^450^ CD4 | RPA-T4 | Tonbo Biosciences |  |
| FITC CD19 | HIB19 | BD Biosciences |  |
|  |  |  |  |
